# Predicting platinum-resistance in advanced ovarian cancer: defining patient and disease characteristics to improve treatment approaches at initiation of treatment or earlier in the course of the disease

**DOI:** 10.3389/fonc.2026.1765220

**Published:** 2026-02-13

**Authors:** Dana M. Chase, Gregory Patton, Srinivas Annavarapu, Junxin Shi, Elizabeth Szamreta, Matthew Monberg

**Affiliations:** 1 Division of Gynecologic Oncology, David Geffen School of Medicine at University of California, Los Angeles (UCLA), Los Angeles, CA, United States; 2 Ontada, Boston, MA, United States; 3 Merck & Co., Inc., Rahway, NJ, United States

**Keywords:** ovarian cancer, overall survival, platinum resistance, platinum-based chemotherapy, platinum sensitivity, progression-free survival, real-world data

## Abstract

**Introduction:**

This study aimed to describe differences in characteristics and outcomes between patients with platinum sensitive or platinum resistant advanced ovarian cancer in a community oncology setting.

**Methods:**

This was a retrospective study of adult patients with Stage III/IV ovarian cancer who received 4+ cycles of a front-line platinum-based regimen between 01/01/2017 and 06/30/2021 (followed until 12/31/2021) within The US Oncology Network. Platinum sensitive or resistant patients were included while refractory patients were excluded. Structured and chart review data were used. Multivariable logistic regression assessed factors associated with platinum sensitivity vs. resistance.

**Results:**

A total of 108 platinum sensitive and 34 resistant patient charts were reviewed. In addition to platinum-based chemotherapy, 19% of patients also initiated bevacizumab as part of their front-line regimen. Most patients were White (63%) and diagnosed at Stage IIIC (55%) or IV (33%). ECOG performance status scores were 0/1 for 47% and 2+ for 20% of patients while BRCA mutations were observed among 7.7%. Sensitive vs. resistant patients were younger (p=0.045) and fewer were obese (p=0.011). Stage IV disease (odds ratio [OR]=3.6, p=0.009) was associated with significantly higher odds of platinum resistance after front-line treatment compared to Stage III, while obesity (OR = 2.6, p=0.067) and ECOG 2+ (OR = 2.9, p=0.057) were non-significantly associated with higher odds of resistance. Median overall survival was not reached in sensitive patients, and was 16.7 months in resistant patients. Median progression-free survival in sensitive and resistant patients was 19.6 and 7.9 months, respectively.

**Discussion:**

When exploring factors associated with response to front-line platinum-based treatment of ovarian cancer, Stage IV disease was significantly associated with resistance. As platinum resistant patients have poorer survival and fewer treatment options, it is important to further define the biology and appropriate initial management of these patients.

## 1 Introduction

Approximately 55% of patients with ovarian cancer have distant metastases at diagnosis and another 20% have regional lymph node involvement (1). While most patients with advanced (International Federation of Gynecology and Obstetrics Stage II–IV) ovarian cancer respond to front-line (1L) treatment with debulking surgery and platinum-based chemotherapy, approximately 70% will relapse (2, 3). Patients considered platinum-refractory (progressing during therapy or within 4 weeks after the last dose) or platinum-resistant (relapse within 6 months of platinum-based therapy) have poor prognosis with an expected survival of less than 12 months (4). Patients with a response duration longer than 6 months after platinum therapy are categorized as platinum-sensitive (5, 6).

Guidelines recommend targeted therapies in the maintenance setting among patients with advanced ovarian cancer (3, 7, 8). Bevacizumab, a monoclonal antibody angiogenesis inhibitor, is an option in both newly diagnosed, platinum-sensitive relapsed, or platinum-resistant relapsed ovarian cancer (6, 9, 10). Bevacizumab may be used as a maintenance regimen among patients with a response to platinum-based regimens incorporating bevacizumab. Additionally, poly ADP-ribose polymerase inhibitors (PARPi) targeting single strand break repair and base excision repair pathways are recommended in maintenance settings (7). However, complete or partial response to platinum is a pre-requisite for use of maintenance PARPi (2, 11–13).

Assessment of a patient’s suitability for PARPi therapy includes multiple factors including Breast Cancer Susceptibility Gene (BRCA) mutation status and response to platinum chemotherapy. Several studies have identified factors associated with improved olaparib response, including BRCA mutation, platinum sensitivity, heterozygosity, and hormone receptor expression (14–17). Add-on therapies were also associated with improved response, including anti-angiogenic agents, immunotherapy, and deoxyribose nucleic acid-damage repair targeting agents (18–26). However, these studies were all randomized controlled trials.

Unfortunately, we have yet to define and/or predict those patients on maintenance therapy who will recur within a period that signals platinum resistance. The prediction of platinum sensitivity vs. resistance would allow for better design of treatment approaches in the neoadjuvant/adjuvant settings, different sequencing of treatments, and better-defined survivorship/surveillance strategies. The current study addresses an unmet need to understand factors associated with platinum sensitivity or resistance, including patient characteristics and treatment patterns, in the real-world setting.

This study aimed to describe patient characteristics, treatment patterns, and outcomes among patients with ovarian cancer, stratified by platinum-sensitive and resistance subgroups, and to identify factors associated with resistance to platinum therapy, in a United States (US) community oncology setting.

## 2 Materials and methods

### 2.1 Study design and patient population

This was a retrospective study of adult female patients with advanced (stage III/IV) ovarian, primary peritoneal or fallopian tube cancer who received at least four cycles of a platinum-based regimen in the 1L setting during 1 January 2017 to 30 June 2021 and had at least two visits within The US Oncology Network (The Network). Prior to initiation of the study, the study was reviewed by the US Oncology, Inc. Institutional Review Board, who approved its exemption status and waiver of consent.

Eligible patients were followed longitudinally until 31 December 2021, last patient record, or date of death, whichever occurred first. The end of the study period is after US Food and Drug Administration approvals of PARPi for 1L maintenance treatment of patients with advanced epithelial ovarian cancer in partial or complete response to platinum treatments. Patients who were platinum resistant or sensitive were included while refractory patients and those with a history of platinum use prior to index were excluded from further analyses. Patients were also excluded if, during the study observation period, they were enrolled in a clinical trial, had a diagnosis of another primary cancer, or received an anticancer treatment for a primary cancer other than ovarian cancer.

The index date was date of 1L platinum regimen initiation for ovarian cancer during the study identification period. In case of neoadjuvant platinum treatment followed by interval debulking surgery, the neoadjuvant treatment together with adjuvant treatment was considered as one line of therapy. Platinum resistance was defined by relapse within 30 days to <6 months of discontinuing 1L platinum after receiving 4 to 6 cycles, or relapse anytime within 6 months after receiving ≥7 cycles. Refractory patients relapsed during or within <30 days of discontinuing 4 to 6 cycles of treatment with 1L platinum. Platinum sensitive patients had no evidence of relapse within 6 months of discontinuing 1L platinum.

### 2.2 Data source

Study data were sourced from the iKnowMed electronic health records (EHR) database, supplemented by death dates from the Social Security Administration’s Limited Access Death Master File. The Network includes 1, 400 affiliated physicians operating in over 500 sites across states and treats approximately 1.2 million US cancer patients annually (5). iKnowMed captures data on outpatient medical oncology care for patients treated across the US.

Structured data were collected via programmatic queries of the iKnowMed EHR and supplemented using unstructured data from targeted chart abstraction. The structured data were used for initial identification and categorization of patients into platinum sensitivity cohorts. Initiation of next treatment and death were used as proxies for relapse. Chart review data were used to confirm relapse status through provider-assessed relapse dates (**Supplementary Table 1**). All chart reviewers have extensive experience in both abstraction and oncology, and undergo comprehensive, study-specific training. Quality metrics are reviewed during and at completion of chart abstraction with a targeted accuracy threshold of 90%; any scores below this threshold prompt additional quality reviews and necessary corrections.

### 2.3 Outcomes

Overall survival (OS) was defined as the interval between index event and date of death (any cause) as documented in the Limited Access Death Master File or in the iKnowMed EHR.

Real-world progression-free survival (rwPFS) was measured from initiation of 1L platinum-based chemotherapy to the earliest date of progression or date of death due to any cause. Real-world progression was assessed during chart review and was based solely on explicit provider documention of progression in progress notes. In analyses of OS and rwPFS, patients who did not experience events during the study observation period were censored at their last visit date.

### 2.4 Statistical analysis

Descriptive analyses were conducted to evaluate demographic, clinical and treatment characteristics overall, and for each study cohort by platinum sensitivity status. OS and rwPFS were analyzed using Kaplan-Meier (KM) methods with 95% confidence intervals (CI). Age at index, body mass index (BMI) category, stage at initial diagnosis, Eastern Cooperative Oncology Group performance status (ECOG PS), Charlson Comorbidity Index (CCI), and first-line treatment were chosen *a priori* based on clinical relevance and included in a multivariable logistic regression analysis of factors associated with 1L platinum resistance. The model included a “missing” category for ECOG PS in order to retain those patients with missing ECOG data in the analysis.

## 3 Results

### 3.1 Patient characteristics

A total of 401 patients met eligibility criteria and were categorized into three cohorts based on platinum sensitivity status: refractory (n=10), platinum resistant (n=89), and platinum sensitive (n=256). There were 46 patients whose platinum sensitivity status was not defined (1L platinum treatment was ongoing or the patients were lost to follow up). The target sample size for chart abstraction was 150 charts, including a random sample of 75 patients from each of the resistant and sensitive cohorts. However, after verifying eligibility during chart review, the final analytic cohort consisted of 142 patients, 108 in the platinum-sensitive and 34 in the platinum-resistant cohorts (**Supplementary Figure 1**).

In the overall cohort, the majority of patients were White (63.4%), and their median (interquartile range [IQR]) age was 70 (60, 77) years (**Table 1**). The highest proportion of patients overall were of normal BMI (37.3%). Half (50.0%) of patients were treated at practice locations in the West, followed by the South (36.6%). The highest proportion of patients were diagnosed at Stage IIIC (54.9%), with 33.1% diagnosed at Stage IV (**Table 2**). Most patients overall had epithelial ovarian cancer (82.4%).

**Table 1:** Demographic characteristics of patients with ovarian cancer initiating 1L platinum treatment, by platinum sensitivity status.

Demographic characteristic	Overall (N = 142)	Platinum resistant (N = 34)	Platinum sensitive (N = 108)	P-value
Age at index*, years				0.0447
N	142	34	108	.
Mean (SD)	68.2 (12.0)	72.2 (10.2)	67.0 (12.3)	.
Median (IQR)	70 (60, 77)	71 (65, 78)	69 (59, 75)	.
Age group, n (%)				0.0843
<65 years	51 (35.9)	8 (23.5)	43 (39.8)	.
≥65 years	91 (64.1)	26 (76.5)	65 (60.2)	.
Race, n (%)				0.4471
Caucasian/White	90 (63.4)	18 (52.9)	72 (66.7)	.
Black/African American	10 (7.0)	4 (11.8)	6 (5.6)	.
Other	10 (7.0)	2 (5.9)	8 (7.4)	.
Not documented	32 (22.5)	10 (29.4)	22 (20.4)	.
BMI, n (%)				0.0108
Underweight	6 (4.2)	4 (11.8)	2 (1.9)	.
Normal	53 (37.3)	10 (29.4)	43 (39.8)	.
Overweight	37 (26.1)	5 (14.7)	32 (29.6)	.
Obese	40 (28.2)	14 (41.2)	26 (24.1)	.
Not documented	6 (4.2)	1 (2.9)	5 (4.6)	.
Practice location, n (%)				0.7472
Midwest	16 (11.3)	5 (14.7)	11 (10.2)	.
Northeast	3 (2.1)	1 (2.9)	2 (1.9)	.
South	52 (36.6)	12 (35.3)	40 (37.0)	.
West	71 (50.0)	16 (47.1)	55 (50.9)	.

BMI, body mass index; IQR, interquartile ratio; SD, standard deviation.

*The index date was defined as the initiation date of the 1L platinum regimen indicated for ovarian cancer during the study identification period. Unless otherwise specified, baseline demographic and clinical characteristics were assessed at the closest date within ±30 days of index date.

**Table 2:** Clinical characteristics of patients with ovarian cancer initiating 1L platinum treatment, by platinum sensitivity status.

Clinical characteristic	Overall(N = 142)	Platinum resistant(N = 34)	Platinum sensitive(N = 108)	P-value
Stage at initial ovarian cancer diagnosis, n (%)				0.1505
Stage III (NOS)	3 (2.1)	0 (0.0)	3 (2.8)	.
Stage IIIA1	5 (3.5)	1 (2.9)	4 (3.7)	.
Stage IIIA2	3 (2.1)	0 (0.0)	3 (2.8)	.
Stage IIIB	6 (4.2)	1 (2.9)	5 (4.6)	.
Stage IIIC	78 (54.9)	14 (41.2)	64 (59.3)	.
Stage IV (NOS)	28 (19.7)	11 (32.4)	17 (15.7)	.
Stage IVA	5 (3.5)	3 (8.8)	2 (1.9)	.
Stage IVB	14 (9.9)	4 (11.8)	10 (9.3)	.
ECOG performance status, n (%) (using +/- 30 days window)				0.0348
0-1	67 (47.2)	12 (35.3)	55 (50.9)	.
2+	29 (20.4)	11 (32.4)	18 (16.7)	.
Not documented	46 (32.4)	11 (32.4)	35 (32.4)	.
Histology, n (%)				.
Epithelial	117 (82.4)	26 (76.5)	91 (84.3)	.
Other	11 (7.7)	3 (8.8)	8 (7.4)	.
Not documented	14 (9.9)	5 (14.7)	9 (8.3)	.
Surgery prior to index treatment				
Patients with available data	55 (38.7)	11 (32.3)	44 (40.7)	
Macroscopic residual disease, n (%)				0.4429
No	41 (74.5)	7 (63.6)	34 (77.3)	
Yes	14 (25.5)	4 (36.4)	10 (22.7)	
Surgery after index treatment prior to 2L/progression				
Patients with available data	40 (28.2)	5 (14.7)	35 (32.4)	
Macroscopic residual disease, n (%)				0.5066
No	35 (87.5)	4 (80.0)	31 (88.6)	
Yes	5 (12.5)	1 (20.0)	4 (11.4)	
BRCA1 status, n (%)				1.0000
Negative	81 (57.0)	21 (61.8)	60 (55.6)	.
Positive	6 (4.2)	1 (2.9)	5 (4.6)	.
VUS	1 (0.7)	0 (0.0)	1 (0.9)	.
Not documented	54 (38.0)	12 (35.3)	42 (38.9)	.
BRCA2 status, n (%)				0.1397
Negative	81 (57.0)	22 (64.7)	59 (54.6)	.
Positive	6 (4.2)	0 (0.0)	6 (5.6)	.
Not documented	55 (38.7)	12 (35.3)	43 (39.8)	.
BRCA status, n (%)				0.2785
Negative	77 (54.2)	21 (61.8)	56 (51.9)	.
Positive	11 (7.7)	1 (2.9)	10 (9.3)	.
Not documented	54 (38.0)	12 (35.3)	42 (38.9)	.
Ca-125, n (%)				.
Normal (0–46 u/mL)	19 (13.4)	1 (2.9)	18 (16.7)	.
Elevated (>46 u/mL)	103 (72.5)	28 (82.4)	75 (69.4)	.
Not documented	20 (14.1)	5 (14.7)	15 (13.9)	.
Homologous recombination deficiency status, n (%)				.
Negative	7 (4.9)	1 (2.9)	6 (5.6)	.
Positive	8 (5.6)	1 (2.9)	7 (6.5)	.
Not documented	127 (89.4)	32 (94.1)	95 (88.0)	.
Charlson comorbidities within 6 months prior to initiation of 1L platinum (>5% of patients), n (%)				.
Hypertension	61 (43.0)	14 (41.2)	47 (43.5)	0.8099
Diabetes	19 (13.4)	6 (17.6)	13 (12.0)	0.4020
Chronic Obstructive Pulmonary Disease	17 (12.0)	4 (11.8)	13 (12.0)	1.0000
Cerebrovascular Disease	11 (7.7)	2 (5.9)	9 (8.3)	1.0000
Acute MI or history of MI	7 (4.9)	2 (5.9)	5 (4.6)	0.6727
Rheumatologic Disease/Connective Tissue Disease	6 (4.2)	3 (8.8)	3 (2.8)	0.1487
Diabetes with sequelae	5 (3.5)	3 (8.8)	2 (1.9)	0.0890
Charlson Comorbidity Index, n (%)				0.6898
0	90 (63.4)	22 (64.7)	68 (63.0)	.
1-2	43 (30.3)	9 (26.5)	34 (31.5)	.
3+	9 (6.3)	3 (8.8)	6 (5.6)	.

BRCA, BReast CAncer Susceptibility gene; ECOG, Eastern Cooperative Oncology Group; MI, myocardial infarction; NOS, not otherwise specified; VUS, variant of uncertain significance.

Platinum sensitive patients were significantly younger than resistant patients (median [IQR] 69 (59, 75) vs. 71 [65, 78]; p=0.045]. BMI was significantly different between the cohorts (p=0.011); more sensitive vs. resistant patients were normal weight (39.8% vs. 29.4%) or overweight (29.6% vs. 14.7%), and fewer were obese (24.1% vs. 41.2%). A higher proportion of the resistant vs. sensitive cohort had Stage IV disease at initial diagnosis (53.0% vs 26.9%, respectively). ECOG PS scores were significantly different between the sensitive and resistant cohorts; 16.7% vs. 32.4% had ECOG PS 2+ in sensitive vs. resistant patients (p=0.0348). Among patients who had surgery, a higher proportion of the sensitive cohort underwent primary debulking surgery prior to index treatment (40.7% vs. 32.4% of the resistant cohort) and interval debulking surgery after index treatment before initiating second-line (2L) or having disease progression (32.4% vs 14.7% of the resistant cohort). There was no statistically significant difference between the proportions of BRCA-negative sensitive (51.9%) and resistant (61.8%) patients.

The CCI was similar across the platinum sensitivity cohorts, and was 0 for 63.4% of the overall study population. While 9.3% and 2.9% of the platinum sensitive and resistant cohorts were BRCAm, respectively, 38.9% and 35.3% among the respective cohorts had no documentation. Homologous recombination deficiency status, as entered by providers in the electronic health record database, was not documented among nearly all patients (89.4% overall). Ca-125 was elevated among 69.4% and 82.4% of the sensitive and resistant cohorts, respectively.

### 3.2 Treatment patterns

Most patients in both the platinum sensitive (67%) and resistant (74%) cohorts received carboplatin with paclitaxel as their initial platinum regimen, and nearly all patients received a regimen containing both carboplatin and paclitaxel with or without other agents (**Table 3**, **Supplementary Table 2**).

**Table 3:** Treatment patterns of patients with ovarian cancer initiating 1L platinum treatment, by platinum sensitivity status.

Treatment	Overall (N = 142)	Platinum resistant (N = 34)	Platinum sensitive (N = 108)
LOT1 regimens – n (% of LOT1 patients)			
Platinum-based chemotherapy regimens	115 (81.0%)	30 (88.2%)	85 (78.7%)
Platinum + bevacizumab-based chemotherapy regimens	27 (19.0%)	4 (11.8%)	23 (21.3%)
LOT1 time to treatment discontinuation, months			
N	142	34	108
Mean (SD)	3.7 (2.0)	4.7 (3.1)	3.4 (1.3)
Median (IQR)	3.7 (2.6, 4.2)	4 (3.5, 4.9)	3.6 (2.1, 4.1)
Patients who did not proceed after LOT1	57	7	50
Patient died after LOT1, n (% of patients who did not proceed after LOT1)	13 (22.8%)	7 (100.0%)	6 (12.0%)
Patient did not proceed after LOT1 and without evidence of death, n (% of patients who did not proceed after LOT1)	44 (77.2%)	0 (0.0%)	44 (88.0%)
LOT2 – n (% of total study population)	85 (59.9%)	27 (79.4%)	58 (53.7%)
LOT2 regimens, n (% of LOT2 patients)			
Platinum-based chemotherapy regimens	26 (30.6%)	1 (3.7%)	25 (43.1%)
Platinum and bevacizumab-based regimens	19 (22.4%)	1 (3.7%)	18 (31.0%)
Bevacizumab-based regimens (no platinum)	25 (29.4%)	18 (66.7%)	7 (12.1%)
PARPi-based regimens	3 (3.5%)	1 (3.7%)	2 (3.4%)
Other	12 (14.1%)^a^	6 (22.2%)^b^	6 (10.3%)^c^
LOT2 time to treatment discontinuation, months			
N	85	27	58
Mean (SD)	2.2 (3.6)	1.7 (2.7)	2.4 (3.9)
Median (IQR)	1 (0.3, 2.6)	1 (0.4, 1.9)	1 (0.0, 2.8)
Patients who did not proceed after LOT2	39	9	30
Patient died after LOT2, n (% of patients who did not proceed after LOT2)	18 (46.2%)	7 (77.8%)	11 (36.7%)
Patient did not proceed after LOT2 and without evidence of death, n (% of patients who did not proceed after LOT2)	21 (53.8%)	2 (22.2%)	19 (63.3%)
LOT3 therapy – n (% of total study population)	46 (32.4)	18 (52.9)	28 (25.9)
LOT3 regimens (% of LOT3 patients)			
Platinum-based chemotherapy regimens	15 (32.6%)	6 (33.3%)	9 (32.1%)
Platinum and bevacizumab-based regimens	6 (13.0%)	0 (0.0%)	6 (21.4%)
Bevacizumab-based regimens (no platinum)	8 (17.4%)	3 (16.7%)	5 (17.9%)
PARPi-based regimens	2 (4.3%)	0 (0.0%)	2 (7.1%)
Other	15 (32.6%)	9 (50.0%)	6 (21.4%)
LOT3 time to treatment discontinuation, months			
N	46	18	28
Mean (SD)	3.8 (4.5)	4.2 (6.8)	3.5 (2.2)
Median (IQR)	2.8 (1.2, 4.9)	1.3 (0.5, 4.9)	3.5 (1.7, 5.1)
Patients who did not proceed after LOT3	25	8	17
Patient died after LOT3	15 (60.0)	7 (87.5)	8 (47.1)
Patient did not proceed	10 (40.0)	1 (12.5)	9 (52.9)

IQR, interquartile ratio; LOT1, first line of therapy; LOT2, second line of therapy; LOT3, third line of therapy; SD, standard deviation.

a Other: letrozole, liposomal doxorubicin, paclitaxel, pembrolizumab, pemetrexed.

b Other: liposomal doxorubicin, pemetrexed.

c Other: letrozole, liposomal doxorubicin, paclitaxel, pembrolizumab.

Among the overall study population (n=142), approximately 19% received bevacizumab in 1L in addition to 1L platinum-based chemotherapy, and 8.4% received PARPi-containing regimens (including maintenance), nearly all of them in the platinum sensitive cohort (10.2% of the sensitive cohort, and 2.9% of the resistant cohort). A numerically higher proportion of the sensitive cohort received 1L platinum-based chemotherapy with bevacizumab (21.3%) compared with resistant (11.8%). Most of the resistant cohort (88.2%) received 1L platinum-based chemotherapy without bevacizumab, while the remainder (11.8%) received 1L platinum-based chemotherapy with bevacizumab.

A numerically higher proportion of platinum sensitive patients did not receive 2L treatment after 1L (46.3% vs 20.6% of PR). In 2L, most sensitive patients (74.1%) received another platinum-based chemotherapy with (31.0%) or without bevacizumab (43.1%); only 12.1% received non-platinum-based regimens with bevacizumab. Third-line treatments are presented in **Table 3** and **Supplementary Table 2**.

Median (IQR) time to 1L treatment discontinuation was numerically similar for platinum sensitive and resistant patients: 3.6 (2.1, 4.1) and 4 (3.5, 4.9) months, respectively (**Table 3**, **Supplementary Table 2**).

While higher proportions of the platinum sensitive cohort did not have residual disease after each surgery, differences between the cohorts were not statistically significant, regardless of whether they received primary or interval debulking surgery. Most patients (87.5%) had no residual disease after surgery (**Table 2**).

### 3.3 Predictors of platinum resistance

In the multivariable analysis of factors associated with resistance to 1L platinum treatment, Stage IV disease was associated with significantly higher odds of platinum resistance after 1L compared to Stage III (odds ratio [OR]=3.6, p=0.009) (**Table 4**). Obesity vs. healthy weight (OR = 2.6, p=0.067) and ECOG PS 2+ vs. 0/1 (OR = 2.9, p=0.057) were non-significantly associated with higher odds of resistance after 1L (excluding underweight and unreported BMI categories for low counts). Age, CCI, and 1L bevacizumab use were not associated with resistance in multivariable analysis.

**Table 4:** Multivariable logistic regression analysis of factors associated with 1L platinum resistance.

Covariate	Level	Total	Platinum resistant	Platinum sensitive	Odds ratio (95% CI)	Pairwise P-value	Type III P-value
Age at index*	<65 years (reference)	44	37	7			0.1883
	65+ years	86	64	22	2.04 (0.706, 5.876)	0.1883	
BMI	Normal (reference)	53	43	10			0.0375
	Overweight	37	32	5	0.57 (0.164, 2.004)	0.3844	
	Obese	40	26	14	2.6 (0.934, 7.25)	0.0674	
Stage	Stage III (reference)	87	73	14			0.0087
	Stage IV	43	28	15	**3.59 (1.382, 9.342)**	**0.0087**	
ECOG PS	0-1 (reference)	67	55	12			0.1632
	2+	31	21	20	2.89 (0.969, 8.6)	0.0571	
	Not documented	32	25	7	1.68 (0.519, 5.418)	0.3877	
CCI	0 (reference)	82	64	18			0.4020
	1+	48	38	11	0.66 (0.245, 1.756)	0.4020	
LOT1	Platinum plus Bevacizumab (reference)	25	21	4			0.2241
	Platinum plus others	105	80	25	2.25 (0.609, 8.311)	0.2241	

BMI, body mass index; CCI, Charlson Comorbidity Index; CI, confidence interval; ECOG, Eastern Cooperative Oncology Group Performance Status; LOT1, first line of therapy.

*The index date was defined as the initiation date of the 1L platinum regimen indicated for ovarian cancer during the study identification period. Unless otherwise specified, baseline demographic and clinical characteristics were assessed at the closest date within ±30 days of index date

Bolded values indicate statistical significance (p<0.05).

### 3.4 Overall survival

Median (95% CI) OS was significantly longer in the platinum sensitive cohort compared with the resistant cohort (not reached [NR] [44.6, NR] vs. 16.7 [13.9, 23.6] months, log rank p-value <0.0001) (**Table 5**, **Supplementary Figure 2**). Cox regression analysis showed a greater rate of death associated with platinum resistance (hazard ratio [HR]: 6.28; 95% CI: 3.19, 12.38) after controlling for age, BMI, stage at initial diagnosis, ECOG PS, CCI, and 1L bevacizumab use (**Supplementary Figure 3**, **Supplementary Table 3**).

**Table 5:** Kaplan-Meier analysis of overall survival and real-world progression-free survival of patients with ovarian cancer initiating 1L platinum treatment, by platinum sensitivity status.

Estimate	Overall survival			Real-world progression-free survival		
	Platinum resistant (N = 34)	Platinum Sensitive (N = 108)	Log rank p-value	Platinum resistant (N = 34)	Platinum sensitive (N = 108)	Log rank p-value
Events (%)	29 (85.3)	31 (28.7)	<0.0001	34 (100.0)	70 (64.8)	<0.0001
Mean (SE), months	21.7 (2.4)	41.0 (1.3)		8.7 (0.6)	23.2 (1.0)	
Median (95% CI), months	16.7 (13.9, 23.6)	NR (44.6, NR)		7.9 (7.1, 8.5)	19.6 (17.6, 24.9)	
Q1, Q3	12.6, 27.7	33.2, NR		6.9, 9.7	13.4, NR	
Probability of survival, % (95% CI)						
3 months	NA	NA		NA	NA	
6 months	NA	NA		94.1 (78.5, 98.5)	NA	
9 months	88.2 (71.6, 95.4)	NA		26.5 (13.2, 41.8)	98.1 (92.8, 99.5)	
12 months	79.4 (61.6, 89.6)	99.1 (93.6, 99.9)		8.8 (2.3, 21.1)	87.0 (79.0, 92.1)	
18 months	45.0 (27.6, 61.0)	92.2 (85.0, 96.0)		2.9 (0.2, 13.0)	59.9 (49.8, 68.6)	
24 months	32.2 (17.0, 48.3)	84.7 (75.9, 90.5)		2.9 (0.2, 13.0)	41.9 (32.1, 51.4)	

CI, confidence interval; NA, not applicable; NR, not reached; SE, standard error.

### 3.5 Real-world progression-free survival

Median (95% CI) rwPFS was significantly longer in the platinum sensitive cohort compared with the resistant cohort (19.6 [17.6, 24.9] months vs. 7.9 [7.1, 8.5] months, log rank p-value <0.0001) (**Table 5**, **Supplementary Figure 2**). Cox regression analysis showed resistant patients experienced a greater rate of progression (HR: 16.74; 95% CI: 8.94, 31.35) compared to sensitive patients, after controlling for age, BMI, stage at initial diagnosis, ECOG PS, CCI, and 1L bevacizumab use. Patients diagnosed at Stage IV had a greater rate of progression (HR: 1.58; 95% CI: 1.01, 2.5) compared with Stage III, while obese patients had lower rate of progression compared with normal BMI (HR: 0.51; 95% CI: 0.30, 0.87) (**Supplementary Figure 4**, **Supplementary Table 4**).

## 4 Discussion

This retrospective observational study described characteristics, treatment patterns, and outcomes among patients with stage III/IV ovarian cancer who initiated 1L platinum stratified by platinum sensitivity status. Additionally, patient characteristics associated with response after 1L platinum were evaluated. We observed differences in patient characteristics and outcomes by sensitivity status.

Platinum sensitive patients were younger and had lower ECOG PS scores compared with resistant patients; fewer sensitive patients were obese, had stage IV disease, or had elevated CA-125 compared with resistant patients. The sensitive cohort also had a numerically higher proportion of patients without residual disease after surgery. Similar findings were observed by Cardillo et al., who evaluated the effect of surgical cytoreduction while controlling for response to platinum-based chemotherapy. In their study, platinum sensitive patients were younger, fewer had stage IV disease, and a greater proportion had no residual disease after surgery compared with resistant patients (27). In this study, platinum resistance was associated with more advanced disease stage as well as obesity and worse performance status. A few other studies explored factors associated with platinum resistance. One study observed that neutrophil-lymphocyte ratio >3.38 and advanced stage (III/IV vs I/II) were independent predictive factors for platinum resistance, with adjusted odds ratios of 1.88 and 3.33, respectively (28). Another study evaluating factors associated with survival by platinum sensitivity status observed that neutrophil-lymphocyte ratio ≥3 and systemic inflammatory index ≥730 were associated with worse survival in patients with platinum-sensitive disease (29). Our study did not evaluate association of neutrophil-lymphocyte ratio or systemic inflammatory index with platinum resistance. This study did, however, control for known risk factors, such as age, residual disease at time of surgery, and ECOG PS; ultimately Stage IV disease was the most predictive of platinum resistant disease. This finding suggests tumor biology and metastatic spread are critical factors in the prognosis of ovarian cancer.

Most patients in platinum sensitive and resistant cohorts received carboplatin-based doublet regimens as 1L regimens. A higher proportion of patients in the sensitive cohort received bevacizumab with 1L platinum chemotherapy compared with the resistant cohort; a higher proportion also received PARPi-containing regimens (including maintenance) compared with the resistant cohort.

Median time to treatment discontinuation was similar between platinum sensitive and resistant cohorts while median OS (NR vs. 16.7 months) and rwPFS (19.6 vs. 7.9 months) were longer in the sensitive cohort compared with the resistant cohort. Cox regression analyses confirmed increased risk of death and progression in the resistant cohort compared with sensitive after adjusting for covariates. These findings align with Cardillo et al., who observed a higher median survival of 44.8 months among sensitive patients compared with 18.1 months among resistant patients (27).

Stage IV disease, wild-type BRCA, residual disease after surgery, and interval debulking surgery (relative to primary debulking surgery), were previously found to be associated with worse survival in patients with advanced ovarian cancer (30). Further, Chase et al., found that patients without these risk factors had median overall survival of 87.8 months compared with 18.5 months among patients with all four risk factors (31). However, these studies did not explore factors associated with platinum resistance.

### 4.1 Strengths and limitations

Limitations of this study include its small sample size, particularly the sparse number of patients in the platinum-resistant subgroup, which limited the number of candidate variables for inclusion in the multivariable model for platinum resistance, and likely reduced our statistical power to detect associations. Biomarker data, including BRCA and HRD status, were not available for a substantial proportion (~35-40%) of patients, precluding their inclusion in further analyses. ECOG performance status was also missing for about a third of patients, suggesting that an important prognostic variable is still not routinely documented in oncology practice. For the multivariable analysis, the ECOG variable included a “missing” category in order to retain those patients in the model. This method assumes missingness is random, which, while unverifiable, is supported by the lack of association between this category, compared to those with ECOG 0-1, and the outcome (OR: 1.7; 95% CI: 0.52, 5.42); by comparison, those with ECOG 2+ had an elevated odds of platinum sensitivity that approached statistical significance (OR: 2.9; 95% CI: 0.97, 8.6).

Results from this study must be interpreted in the context of strengths and limitations of real-world research. This study used EHR data from a large network of US community oncology practices reflecting real-world treatment patterns and outcomes as opposed to clinical trials. Data are entered into the iKnowMed EHR primarily for clinical practice purposes, not for research. These data are subject to coding errors of omission or commission, which may introduce misclassification bias of certain diagnoses or events. Some study variables are not complete across the entire study population. Visibility into services and treatments received outside The Network may be limited. Practices within The Network follow evidence-based guidelines that may differ from other community oncology clinics, which may limit generalizability.

## 5 Conclusions

When exploring factors associated with response to 1L platinum-based treatment of ovarian cancer adjusting for other factors, stage IV disease was significantly associated with platinum resistance, while obesity and poor ECOG PS score were associated with greater odds of resistance. Real-world outcomes were significantly better among platinum sensitive patients, highlighting a need to explore early identification and initial treatments for resistant patients. As platinum resistant patients have poorer survival and fewer treatment options, it is important to further define the biology and appropriate initial management of these patients.

## Data Availability

The datasets analyzed for this study are restricted by The US Oncology Research Institutional Review Board in order to protect patient privacy. As a result, data used to support the findings of this study have not been made available.
