# Supplementary material for: Predicting platinum-resistance in advanced ovarian cancer: defining patient and disease characteristics to improve treatment approaches at initiation of treatment or earlier in the course of the disease
Source: Front Oncol. 2026 Feb 13;16:1765220. doi: 10.3389/fonc.2026.1765220 (PMC12946934; doi:10.3389/fonc.2026.1765220)
Supplement: Supplementary file 1 [file Table1.docx]

Supplementary Material

# Supplementary Figures and Tables

## Supplementary Figures

**
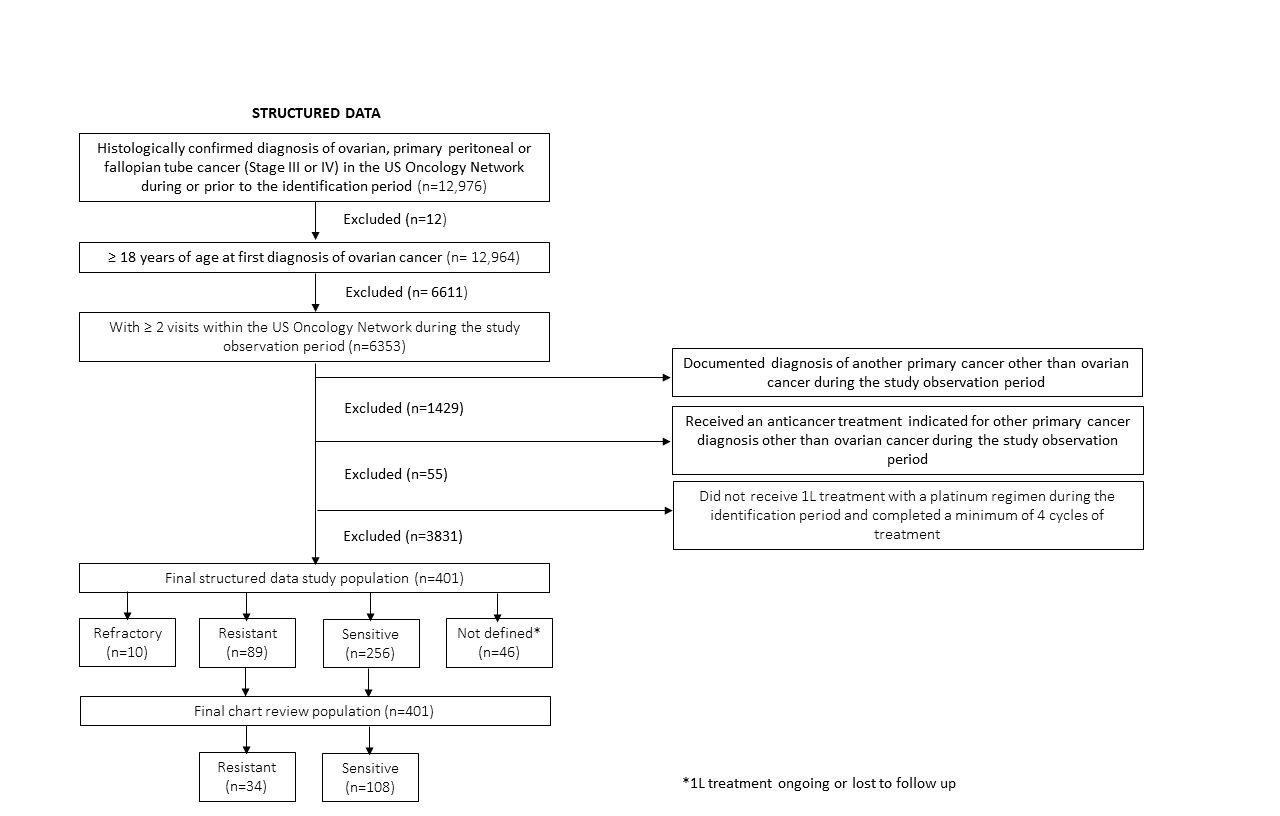
**

**Supplementary Figure 1.** Study Attrition

**
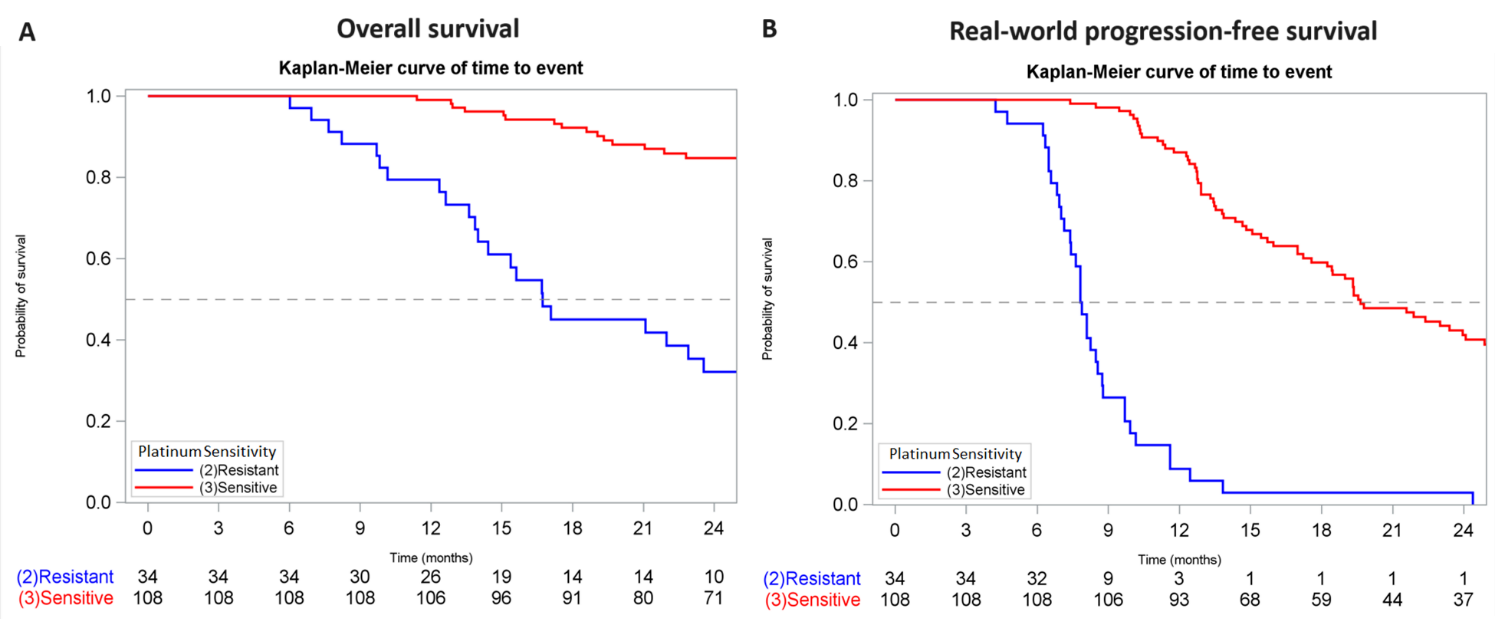
**

**Supplementary Figure 2.** Kaplan-Meier Analysis of Overall Survival and Real-world Progression-free Survival of Patients with Ovarian Cancer Initiating 1L Platinum Treatment, by Platinum Sensitivity Status


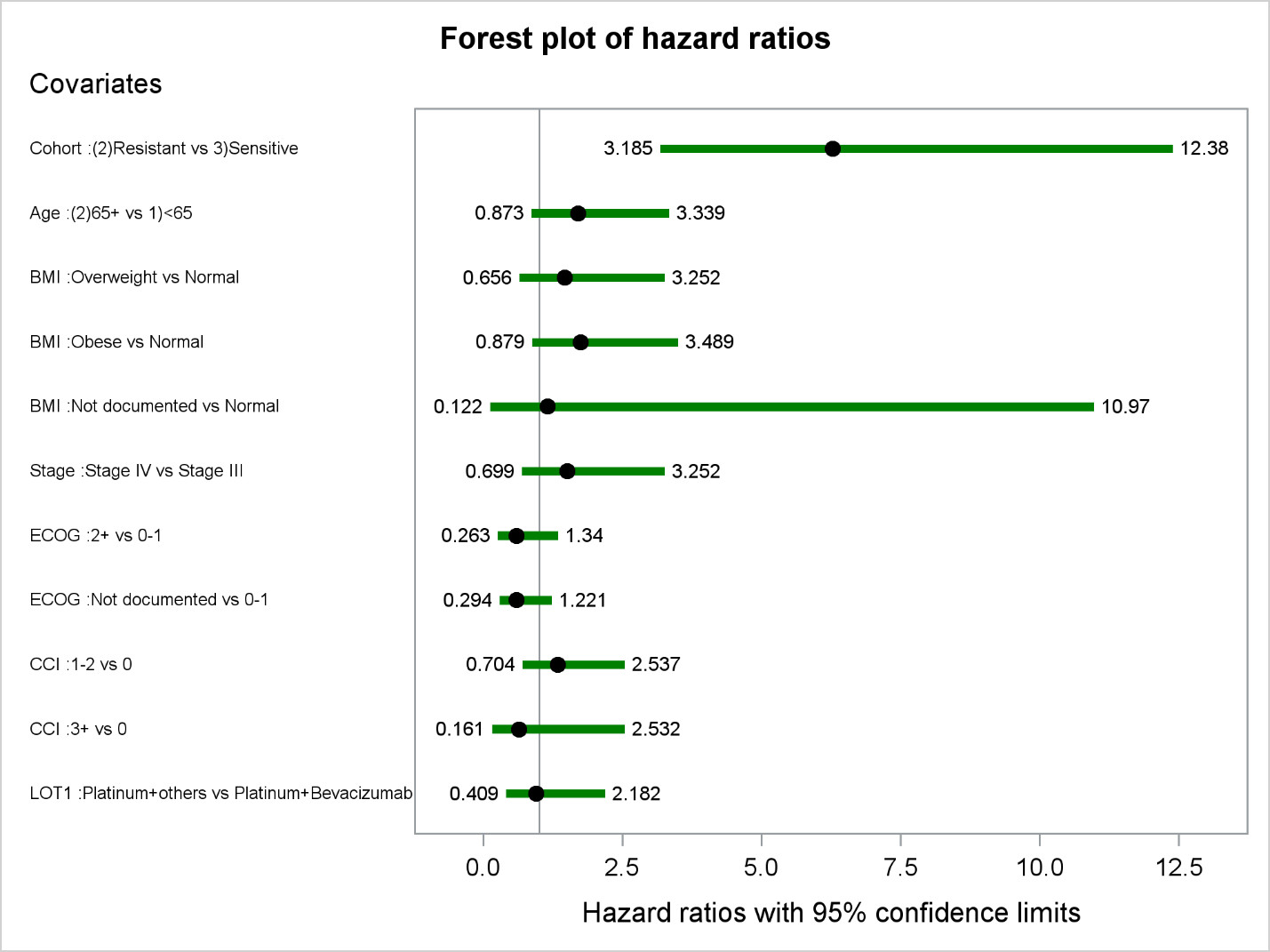


**Supplementary Figure 3.** Forest Plot of Hazard Ratios for Overall Survival

*Legend: BMI, body mass index; CCI, Charlson Comorbidity Index; ECOG, Eastern Cooperative Oncology Group; LOT1, first line of therapy*


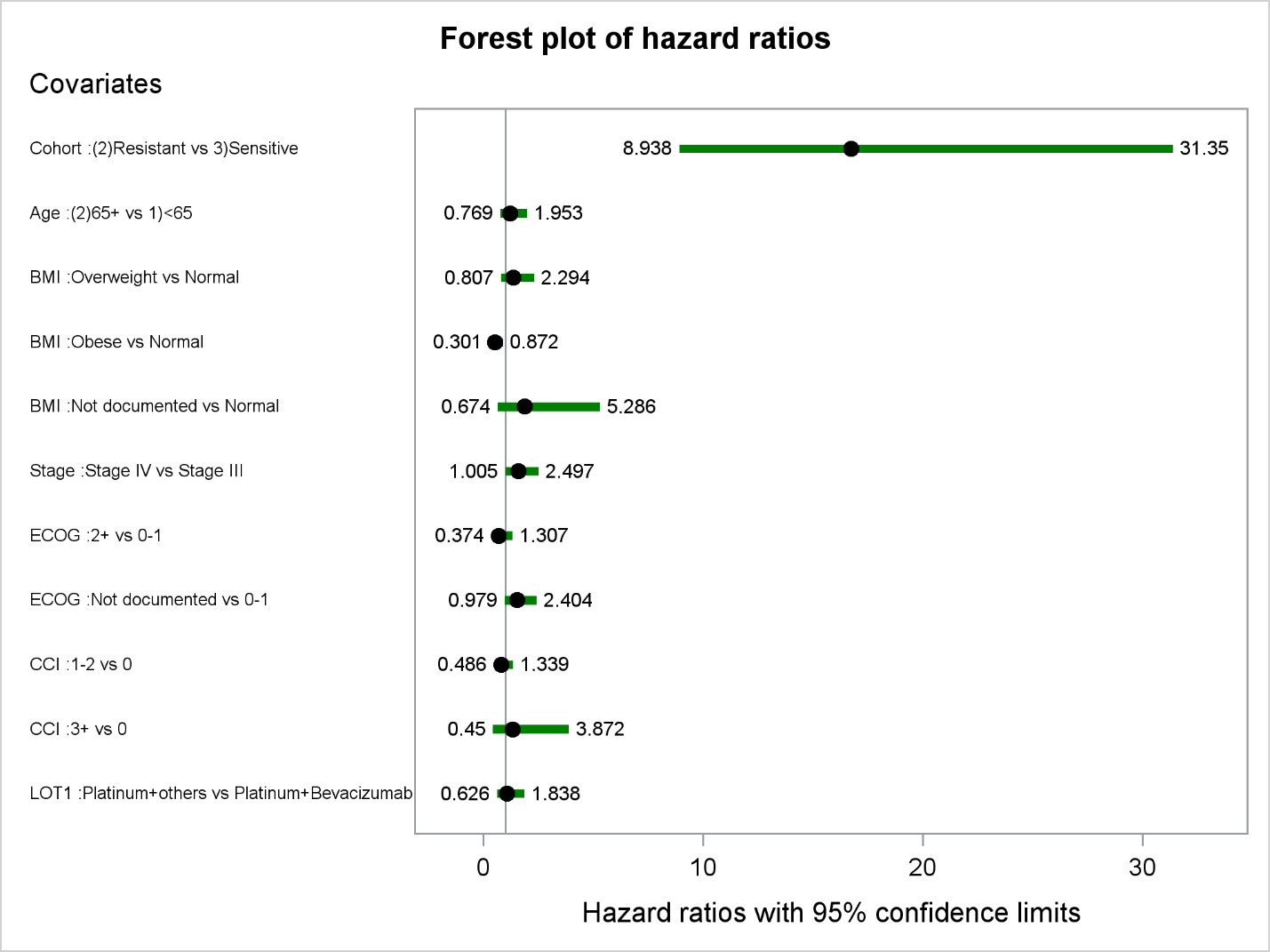


**Supplementary Figure 4.** Forest Plot of Hazard Ratios for Real-world Progression-free Survival

*Legend: BMI, body mass index; CCI, Charlson Comorbidity Index; ECOG, Eastern Cooperative Oncology Group; LOT1, first line of therapy*

## Supplementary Tables

**Supplementary Table 1.** **Definitions of Platinum Sensitivity Categories Determined using Structured Data**

| **Term** | **No. of LOT1 platinum cycles** | **Duration from LOT1 platinum discontinuation to LOT2/death^a^** | **Duration from LOT1 platinum discontinuation to last contact date** |
| --- | --- | --- | --- |
| Refractory | 4 to 6 | <30 days |  |
| PR | 4 to 6 | 30 days to <6 months |  |
|  | 7+ cycles | <6 months |  |
| PST | 4 to 6 | 6 months + |  |
|  | 4 to 6 | No record of LOT2/death | 6 months + |
|  | 7+ cycles | 6 months + |  |
|  | 7+ cycles | No record of LOT2/death | 6 months + |
| Ongoing/ lost to follow up | 4 to 6 | No record of LOT2/death | <6 months |
|  | 7+ cycles | No record of LOT2/death | <6 months |
| LOT1, first line of therapy; LOT2, second line of therapy; PR, platinum resistant; PST, platinum sensitive | | | |
| ^a^ Duration to LOT2 initiation / death / last contact date was calculated from last date of LOT1 platinum administration. Duration between end of LOT1 and initiation of LOT2 was calculated from date of last administration of LOT1 platinum; maintenance treatments were not considered. | | | |

Supplementary Table 2. Treatment Patterns of Patients with Ovarian Cancer Initiating 1L Platinum Treatment, by Platinum Sensitivity Status

| **Analysis Variable** | **Overall (N=142)** | **Platinum Resistant (N=34)** | **Platinum Sensitive (N=108)** |
| --- | --- | --- | --- |
| **Patients initiating LOT1 – n (% of total study population)** | **142 (100.0)** | **34 (100.0)** | **108 (100.0)** |
| **LOT1 regimens – n (% of LOT1 patients)** |  |  |  |
| Carboplatin+Paclitaxel | 97 (68.3) | 25 (73.5) | 72 (66.7) |
| Carboplatin+Paclitaxel>Olaparib | 5 (3.5) | 1 (2.9) | 4 (3.7) |
| Bevacizumab+Carboplatin+Paclitaxel>Bevacizumab | 4 (2.8) | 1 (2.9) | 3 (2.8) |
| Carboplatin+Paclitaxel>Bevacizumab | 4 (2.8) | 1 (2.9) | 3 (2.8) |
| Bevacizumab+Carboplatin+Paclitaxel | 3 (2.1) | 1 (2.9) | 2 (1.9) |
| Bevacizumab+Carboplatin+Paclitaxel>Olaparib | 3 (2.1) | 0 (0.0) | 3 (2.8) |
| Bevacizumab-awwb+Carboplatin+Paclitaxel | 3 (2.1) | 0 (0.0) | 3 (2.8) |
| Bevacizumab+Carboplatin+Paclitaxel>Bevacizumab-awwb | 2 (1.4) | 0 (0.0) | 2 (1.9) |
| Bevacizumab-awwb+Carboplatin+Paclitaxel>Bevacizumab-awwb | 2 (1.4) | 0 (0.0) | 2 (1.9) |
| Cisplatin+Paclitaxel | 2 (1.4) | 1 (2.9) | 1 (0.9) |
| Bevacizumab+Carboplatin+Paclitaxel>Bevacizumab>Bevacizumab-awwb | 1 (0.7) | 0 (0.0) | 1 (0.9) |
| Bevacizumab+Carboplatin+Paclitaxel>Niraparib | 1 (0.7) | 0 (0.0) | 1 (0.9) |
| Bevacizumab+Fluorouracil+Other+Oxaliplatin | 1 (0.7) | 0 (0.0) | 1 (0.9) |
| Bevacizumab-awwb+Carboplatin+Paclitaxel>Bevacizumab+Olaparib>Bevacizumab-awwb+Olaparib | 1 (0.7) | 0 (0.0) | 1 (0.9) |
| Carboplatin | 1 (0.7) | 0 (0.0) | 1 (0.9) |
| Carboplatin+Cisplatin+Paclitaxel | 1 (0.7) | 0 (0.0) | 1 (0.9) |
| Carboplatin+Docetaxel+Paclitaxel | 1 (0.7) | 1 (2.9) | 0 (0.0) |
| Carboplatin+Docetaxel+Paclitaxel>Bevacizumab | 1 (0.7) | 1 (2.9) | 0 (0.0) |
| Carboplatin+Gemcitabine | 1 (0.7) | 1 (2.9) | 0 (0.0) |
| Carboplatin+Oxaliplatin>Niraparib | 1 (0.7) | 0 (0.0) | 1 (0.9) |
| Carboplatin+Paclitaxel>Bevacizumab>Bevacizumab+Letrozole | 1 (0.7) | 0 (0.0) | 1 (0.9) |
| Carboplatin+Paclitaxel>Letrozole | 1 (0.7) | 1 (2.9) | 0 (0.0) |
| Carboplatin+Paclitaxel>Niraparib | 1 (0.7) | 0 (0.0) | 1 (0.9) |
| Carboplatin+Paclitaxel>Pembrolizumab | 1 (0.7) | 0 (0.0) | 1 (0.9) |
| Cisplatin+Docetaxel+Paclitaxel | 1 (0.7) | 0 (0.0) | 1 (0.9) |
| Cisplatin+Gemcitabine+Paclitaxel | 1 (0.7) | 0 (0.0) | 1 (0.9) |
| Cisplatin+Paclitaxel>Tamoxifen | 1 (0.7) | 0 (0.0) | 1 (0.9) |
| **LOT1 time to treatment discontinuation, months** |  |  |  |
| N | 142 | 34 | 108 |
| Mean (SD) | 3.7 (2.0) | 4.7 (3.1) | 3.4 (1.3) |
| Median (IQR) | 3.7 (2.6,4.2) | 4 (3.5,4.9) | 3.6 (2.1,4.1) |
| **LOT1 duration of gaps in treatment, months** |  |  |  |
| N | 142 | 34 | 108 |
| Mean (SD) | 8.3 (12.5) | 3.0 (2.3) | 10.0 (13.8) |
| Median (IQR) | 2.5 (1.3,9.7) | 2.9 (1.1,4.7) | 2.3 (1.4,14.8) |
| **Patients who do not proceed after LOT1** |  |  |  |
| Patient died after LOT1 | 13 (22.8) | 7 (100.0) | 6 (12.0) |
| Patient did not proceed after LOT1 and without evidence of death | 44 (77.2) | 0 (0.0) | 44 (88.0) |
| **LOT2 – n (% of total study population)** | **85 (59.9)** | **27 (79.4)** | **58 (53.7)** |
| **LOT2 regimens – n (% of LOT2 patients)** |  |  |  |
| Bevacizumab+Liposomal doxorubicin | 13 (15.1) | 9 (33.3) | 4 (6.8) |
| Liposomal doxorubicin | 8 (9.3) | 5 (18.5) | 3 (5.1) |
| Carboplatin+Gemcitabine | 6 (7.0) | 0 (0.0) | 6 (10.2) |
| Carboplatin+Liposomal doxorubicin | 6 (7.0) | 0 (0.0) | 6 (10.2) |
| Carboplatin+Paclitaxel | 6 (7.0) | 1 (3.7) | 5 (8.5) |
| Bevacizumab+Carboplatin+Gemcitabine | 4 (4.7) | 0 (0.0) | 4 (6.8) |
| Bevacizumab | 3 (3.5) | 3 (11.1) | 0 (0.0) |
| Bevacizumab+Carboplatin+Gemcitabine>Niraparib | 3 (3.5) | 0 (0.0) | 3 (5.1) |
| Bevacizumab+Carboplatin+Paclitaxel | 2 (2.3) | 0 (0.0) | 2 (3.4) |
| Bevacizumab+Paclitaxel | 2 (2.3) | 1 (3.7) | 1 (1.7) |
| Bevacizumab-awwb+Liposomal doxorubicin | 2 (2.3) | 1 (3.7) | 1 (1.7) |
| Olaparib | 2 (2.3) | 0 (0.0) | 2 (3.4) |
| Paclitaxel | 2 (2.3) | 0 (0.0) | 2 (3.4) |
| Bevacizumab+Carboplatin+Docetaxel | 1 (1.2) | 1 (3.7) | 0 (0.0) |
| Bevacizumab+Carboplatin+Gemcitabine>Bevacizumab>Rucaparib | 1 (1.2) | 0 (0.0) | 1 (1.7) |
| Bevacizumab+Carboplatin+Gemcitabine>Olaparib | 1 (1.2) | 0 (0.0) | 1 (1.7) |
| Bevacizumab+Carboplatin+Liposomal doxorubicin>Olaparib | 1 (1.2) | 0 (0.0) | 1 (1.7) |
| Bevacizumab+Carboplatin+Paclitaxel>Bevacizumab | 1 (1.2) | 0 (0.0) | 1 (1.7) |
| Bevacizumab+Carboplatin+Paclitaxel>Bevacizumab+Niraparib | 1 (1.2) | 0 (0.0) | 1 (1.7) |
| Bevacizumab+Carboplatin+Paclitaxel>Bevacizumab>Bevacizumab-awwb | 1 (1.2) | 0 (0.0) | 1 (1.7) |
| Bevacizumab+Carboplatin+Paclitaxel>Bevacizumab>Bevacizumab-awwb>Olaparib | 1 (1.2) | 0 (0.0) | 1 (1.7) |
| Bevacizumab+Cisplatin+Gemcitabine | 1 (1.2) | 0 (0.0) | 1 (1.7) |
| Bevacizumab+Cyclophosphamide | 1 (1.2) | 1 (3.7) | 0 (0.0) |
| Bevacizumab+Doxorubicin | 1 (1.2) | 1 (3.7) | 0 (0.0) |
| Bevacizumab+Gemcitabine | 1 (1.2) | 0 (0.0) | 1 (1.7) |
| Bevacizumab+Liposomal doxorubicin>Bevacizumab | 1 (1.2) | 1 (3.7) | 0 (0.0) |
| Bevacizumab+Liposomal doxorubicin>Niraparib | 1 (1.2) | 1 (3.7) | 0 (0.0) |
| Bevacizumab-awwb+Carboplatin+Paclitaxel>Bevacizumab-awwb | 1 (1.2) | 0 (0.0) | 1 (1.7) |
| Carboplatin | 1 (1.2) | 0 (0.0) | 1 (1.7) |
| Carboplatin+Gemcitabine>Olaparib | 1 (1.2) | 0 (0.0) | 1 (1.7) |
| Carboplatin+Liposomal doxorubicin>Niraparib | 1 (1.2) | 0 (0.0) | 1 (1.7) |
| Carboplatin+Paclitaxel>Niraparib>Olaparib | 1 (1.2) | 0 (0.0) | 1 (1.7) |
| Carboplatin+Paclitaxel>Olaparib | 1 (1.2) | 0 (0.0) | 1 (1.7) |
| Cisplatin+Gemcitabine | 1 (1.2) | 0 (0.0) | 1 (1.7) |
| Cisplatin+Gemcitabine>Rucaparib | 1 (1.2) | 0 (0.0) | 1 (1.7) |
| Cisplatin+Paclitaxel | 1 (1.2) | 0 (0.0) | 1 (1.7) |
| Letrozole | 1 (1.2) | 0 (0.0) | 1 (1.7) |
| Niraparib | 1 (1.2) | 1 (3.7) | 0 (0.0) |
| Pembrolizumab | 1 (1.2) | 0 (0.0) | 1 (1.7) |
| Pemetrexed | 1 (1.2) | 1 (3.7) | 0 (0.0) |
| **LOT2 time to treatment discontinuation, months** |  |  |  |
| N | 85 | 27 | 58 |
| Mean (SD) | 2.2 (3.6) | 1.7 (2.7) | 2.4 (3.9) |
| Median (IQR) | 1 (0.3,2.6) | 1 (0.4,1.9) | 1 (0.0,2.8) |
| **LOT2 duration of gaps in treatment, months** |  |  |  |
| N | 85 | 27 | 58 |
| Mean (SD) | 2.2 (3.5) | 1.7 (2.7) | 2.4 (3.9) |
| Median (IQR) | 1 (0.2,2.6) | 1 (0.4,1.9) | 1 (0.0,2.8) |
| **Patients who do not proceed after LOT2** |  |  |  |
| Patient died after LOT2 | 18 (46.2) | 7 (77.8) | 11 (36.7) |
| Patient did not proceed after LOT2 and without evidence of death | 21 (53.8) | 2 (22.2) | 19 (63.3) |
| **LOT3 – n (% of total study population)** | **46 (32.4)** | **18 (52.9)** | **28 (25.9)** |
| **LOT3 regimens – n (% of LOT3 patients)** |  |  |  |
| Cisplatin+Gemcitabine | 5 (10.9) | 4 (22.2) | 1 (3.6) |
| Gemcitabine | 5 (10.9) | 5 (27.8) | 0 (0.0) |
| Liposomal doxorubicin | 5 (10.9) | 2 (11.1) | 3 (10.7) |
| Carboplatin+Gemcitabine | 4 (8.7) | 0 (0.0) | 4 (14.3) |
| Carboplatin+Paclitaxel | 3 (6.5) | 1 (5.6) | 2 (7.1) |
| Bevacizumab | 2 (4.3) | 0 (0.0) | 2 (7.1) |
| Bevacizumab+Carboplatin+Liposomal doxorubicin | 2 (4.3) | 0 (0.0) | 2 (7.1) |
| Bevacizumab+Liposomal doxorubicin | 2 (4.3) | 1 (5.6) | 1 (3.6) |
| Bevacizumab-awwb+Liposomal doxorubicin | 2 (4.3) | 0 (0.0) | 2 (7.1) |
| Paclitaxel | 2 (4.3) | 1 (5.6) | 1 (3.6) |
| Topotecan | 2 (4.3) | 1 (5.6) | 1 (3.6) |
| Bevacizumab+Carboplatin+Gemcitabine | 1 (2.2) | 0 (0.0) | 1 (3.6) |
| Bevacizumab+Carboplatin+Liposomal doxorubicin>Bevacizumab-awwb+Olaparib | 1 (2.2) | 0 (0.0) | 1 (3.6) |
| Bevacizumab+Gemcitabine | 1 (2.2) | 1 (5.6) | 0 (0.0) |
| Bevacizumab+Topotecan | 1 (2.2) | 1 (5.6) | 0 (0.0) |
| Bevacizumab-awwb+Carboplatin+Gemcitabine | 1 (2.2) | 0 (0.0) | 1 (3.6) |
| Bevacizumab-awwb+Carboplatin+Gemcitabine>Bevacizumab-awwb | 1 (2.2) | 0 (0.0) | 1 (3.6) |
| Carboplatin | 1 (2.2) | 1 (5.6) | 0 (0.0) |
| Carboplatin+Liposomal doxorubicin | 1 (2.2) | 0 (0.0) | 1 (3.6) |
| Carboplatin+Paclitaxelprotein-bound | 1 (2.2) | 0 (0.0) | 1 (3.6) |
| Olaparib | 1 (2.2) | 0 (0.0) | 1 (3.6) |
| Paclitaxel protein-bound | 1 (2.2) | 0 (0.0) | 1 (3.6) |
| Rucaparib | 1 (2.2) | 0 (0.0) | 1 (3.6) |
| **LOT3 time to treatment discontinuation, months** |  |  |  |
| N | 46 | 18 | 28 |
| Mean (SD) | 3.8 (4.5) | 4.2 (6.8) | 3.5 (2.2) |
| Median (IQR) | 2.8 (1.2,4.9) | 1.3 (0.5,4.9) | 3.5 (1.7,5.1) |
| **LOT3 duration of gaps in treatment, months** |  |  |  |
| N | 46 | 18 | 28 |
| Mean (SD) | 2.0 (3.8) | 2.2 (5.3) | 1.9 (2.6) |
| Median (IQR) | 1.1 (0.5,1.6) | 0.8 (0.5,1.4) | 1.1 (0.6,2.3) |
| **Patients who do not proceed after LOT3** |  |  |  |
| Patient died after LOT3 | 15 (60.0) | 7 (87.5) | 8 (47.1) |
| Patient did not proceed | 10 (40.0) | 1 (12.5) | 9 (52.9) |

IQR, interquartile range; LOT1, first line of therapy; LOT2, second line of therapy; LOT3, third line of therapy; SD, standard deviation

**Supplementary Table 3. Cox Regression Model of Factors Associated with Overall Survival among Patients with Ovarian Cancer Initiating 1L Platinum Regimens**

| **Covariate** | **Level** | **N** | **Number of events** | **Hazard Ratio (95% CI)** | **Pairwise p-value** | **Type 3 p-value** |
| --- | --- | --- | --- | --- | --- | --- |
| Cohort | Sensitive (reference) | 106 | 31 |  |  | <0.0001 |
|  | Resistant | 30 | 25 | 6.28 (3.19, 12.38) | < 0.0001 |  |
| Age | <65 years (reference) | 49 | 15 |  |  | 0.1180 |
|  | 65+ years | 87 | 41 | 1.71 (0.87, 3.34) | 0.1180 |  |
| BMI | Normal (reference) | 53 | 19 |  |  | 0.4603 |
|  | Overweight | 37 | 16 | 1.46 (0.66, 3.25) | 0.3541 |  |
|  | Obese | 40 | 20 | 1.75 (0.88, 3.49) | 0.1114 |  |
|  | Not documented | 6 | 1 | 1.16 (0.12, 10.97) | 0.8987 |  |
| Stage | Stage III (reference) | 92 | 33 |  |  | 0.2954 |
|  | Stage IV | 44 | 23 | 1.51 (0.7, 3.25) | 0.2954 |  |
| ECOG | 0-1 (reference) | 70 | 33 |  |  | 0.2772 |
|  | 2+ | 32 | 12 | 0.59 (0.26, 1.34) | 0.2092 |  |
|  | Not documented | 34 | 11 | 0.6 (0.29, 1.22) | 0.1585 |  |
| CCI | 0 (reference) | 86 | 33 |  |  | 0.4590 |
|  | 1-2 | 43 | 19 | 1.34 (0.7, 2.54) | 0.3752 |  |
|  | 3+ | 7 | 4 | 0.64 (0.16, 2.53) | 0.5227 |  |
| LOT1 | Platinum plus bevacizumab (reference) | 27 | 9 |  |  | 0.8943 |
|  | Platinum plus others | 109 | 47 | 0.95 (0.41, 2.18) | 0.8943 |  |

BMI, body mass index; CCI, Charlson Comorbidity Index; CI, confidence interval; ECOG, Eastern Cooperative Oncology Group; LOT1, first-line of therapy

Supplementary Table 4. Cox Regression Model of Factors Associated with Real-world Progression-free Survival among Patients with Ovarian Cancer Initiating 1L Platinum Regimens

| **Covariate** | **Level** | **N** | **Number of events** | **Hazard Ratio (95% CI)** | **Pairwise p-value** | **Type 3 p-value** |
| --- | --- | --- | --- | --- | --- | --- |
| Cohort | Sensitive (reference) | 106 | 70 |  |  | <0.0001 |
|  | Resistant | 30 | 30 | 16.74 (8.94, 31.35) | <0.0001 |  |
| Age | <65 (reference) | 49 | 33 |  |  | 0.3918 |
|  | 65+ | 87 | 67 | 1.23 (0.77, 1.95) | 0.3918 |  |
| BMI | Normal (reference) | 53 | 40 |  |  | 0.0137 |
|  | Overweight | 37 | 30 | 1.36 (0.81, 2.29) | 0.2480 |  |
|  | Obese | 40 | 26 | 0.51 (0.3, 0.87) | 0.0138 |  |
|  | Not documented | 6 | 4 | 1.89 (0.67, 5.29) | 0.2269 |  |
| Stage | Stage III (reference) | 92 | 65 |  |  | 0.0477 |
|  | Stage IV | 44 | 35 | 1.58 (1.01, 2.5) | 0.0477 |  |
| ECOG | 0-1 (reference) | 70 | 50 |  |  | 0.0541 |
|  | 2+ | 32 | 23 | 0.7 (0.37, 1.31) | 0.2627 |  |
|  | Not documented | 34 | 27 | 1.53 (0.98, 2.4) | 0.0619 |  |
| CCI | 0 (reference) | 86 | 64 |  |  | 0.5771 |
|  | 1-2 | 43 | 30 | 0.81 (0.49, 1.34) | 0.4059 |  |
|  | 3+ | 7 | 6 | 1.32 (0.45, 3.87) | 0.6127 |  |
| LOT1 | Platinum plus Bevacizumab (reference) | 27 | 17 |  |  | 0.7993 |
|  | Platinum plus others | 109 | 83 | 1.07 (0.63, 1.84) | 0.7993 |  |

BMI, body mass index; CCI, Charlson Comorbidity Index; CI, confidence interval; ECOG, Eastern Cooperative Oncology Group; LOT1, first-line of therapy
